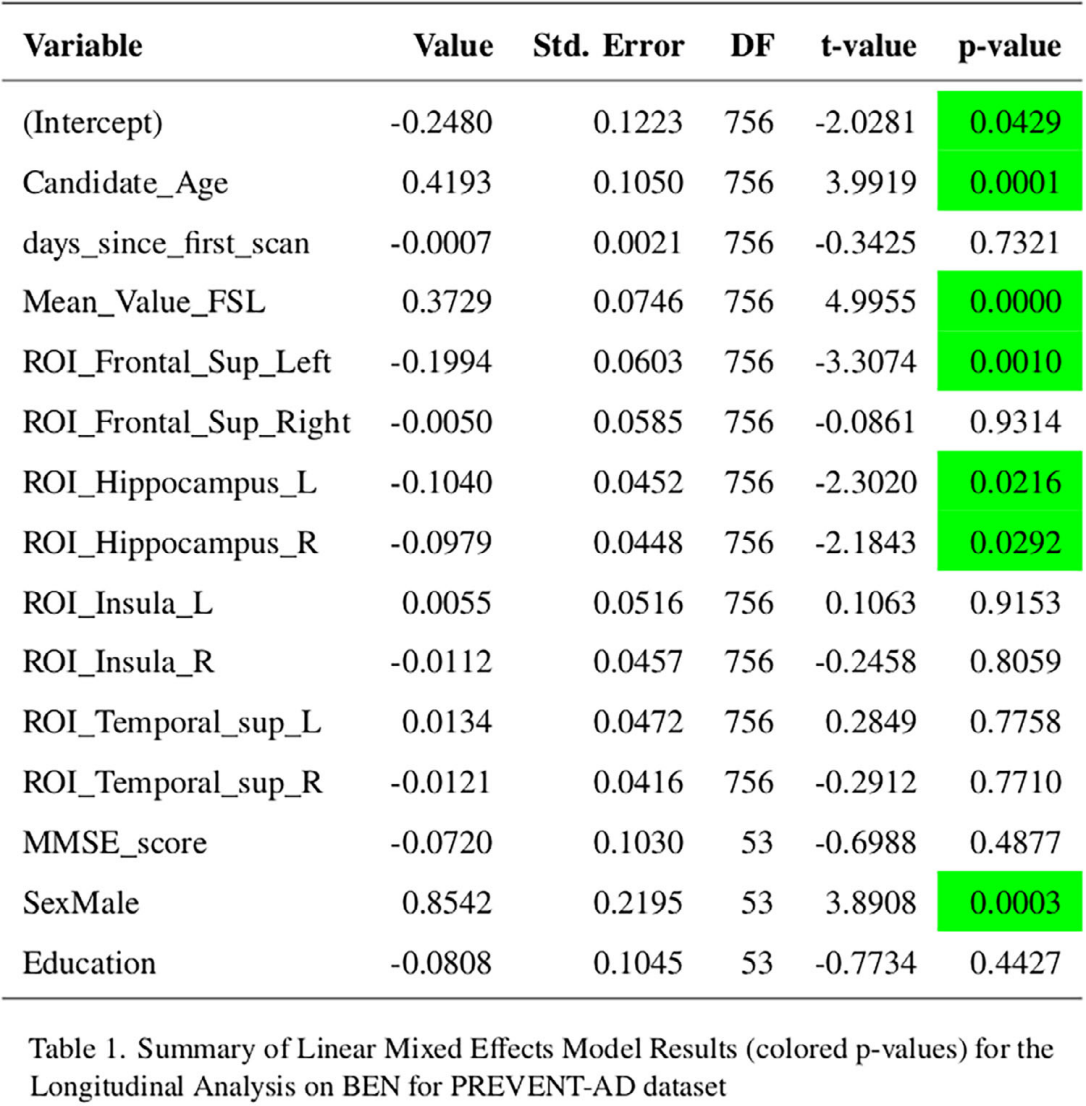# Longitudinal Analysis of Brain Entropy Changes in Alzheimer's Disease

**DOI:** 10.1002/alz70856_103278

**Published:** 2025-12-26

**Authors:** Aldo Camargo, Ze Wang

**Affiliations:** ^1^ University of Maryland of Baltimore, Baltimore, MD, USA

## Abstract

**Background:**

Brain entropy (BEN) measures the level of disorder or irregularity within the brain, and Sample Entropy is often used to quantify the irregularity of time‐series data from brain regions. However, there is limited literature on the longitudinal changes in BEN in Alzheimer's disease (AD). In this study, we used resting‐state fMRI (rsfMRI) data from the PREVENT‐AD dataset to investigate longitudinal changes in Sample Entropy across eight different regions of interest (ROIs).

**Method:**

Data from 122 participants (mean MMSE: 28.82 ± 1.31, age: 67.85 ± 4.85, education: 15.8 ± 2.98 years) were analyzed. Preprocessing steps, including motion correction, slice time correction, normalization, and artifact removal, were performed using fMRIPrep. Sample Entropy was calculated for eight ROIs using in‐house code. Associations of BEN values with MMSE scores, years of education, and gender were assessed using Linear Mixed Models in R.

**Result:**

Table 1 presents the results, showing significant *p*‐values for age, mean whole‐brain BEN, BEN in the Frontal_Sup_Left, Hippocampus_L, Hippocampus_R, and gender (male). Specifically, an increase of one year in participant age was associated with a 0.4193 increase in APS score. For each increment in whole‐brain BEN, the APS score increased by 0.3729. Similarly, an increase of BEN in Frontal_Sup_Left resulted in a reduction of APS score by 0.1994, and a BEN increase in Hippocampus (left and right) decreased APS score by 0.10.

**Conclusion:**

Elevated whole‐brain BEN is associated with higher AD risk, aligning with prior studies linking increased entropy to neuropathological changes like amyloid and tau deposition. However, elevated BEN in specific ROIs, such as Frontal_Sup_Left and hippocampi, may reflect enhanced cognitive adaptability and reduced AD risk. This resilience may result from greater neural variability and complexity, particularly in the hippocampus, where heightened entropy supports memory and consciousness. These findings highlight the dual role of BEN, where global increases suggest AD progression, while region‐specific elevations may indicate protective neural mechanisms.